# Measurement of the effects of the IMAGE MD^®^ skin care regimen on skin surface features via modern imaging technology with the Visia^®^ complexion analysis camera system

**DOI:** 10.3205/iprs000170

**Published:** 2022-11-29

**Authors:** Helga Henseler

**Affiliations:** 1 Klinik am Rhein, Klinik für Plastische und Ästhetische Chirurgie, Düsseldorf, Germany

**Keywords:** IMAGE MD® skin care line, skin surface features, Visia® complexion analysis camera system, objective measurements

## Abstract

**Objective:**

Objective analysis of the effects of a series of skin care products by application of modern imaging technology.

**Method:**

A study was conducted in 25 volunteers who attended a plastic surgical clinic. The cosmeceuticals chosen for investigation were from the IMAGE MD^®^ series provided by the company IMAGE Skincare. Facial images were taken with the Visia^®^ camera system by the company Canfield Scientific. The volunteers stopped their own skin care regimen after the initial facial captures and applied a series of the products for three months. 19 volunteers returned and underwent again facial capture. Eight different skin aspects – spots, wrinkles, skin texture, pores, UV spots, brown spots, red marks and porphyrins – as well as the skin age were determined and analysed.

**Results:**

Overall, the volunteers turned out to appear younger by 1.4 years, however not at a significant level. There was a spread in the results and a tendency for a stronger reduction of the measured skin age in women under 55 years old by 3.2 years. Best effects of the studied product line were obtained for the skin aspects red areas and brown spots. For the criteria spots, texture, pores and UV spots at least two thirds of the comparisons between the two time points, before and after treatment, were positive. In wrinkles and porphyrins, results did not improve. Altogether the majority of the examined skin criteria improved.

**Conclusion:**

The IMAGE MD^®^ product series caused the volunteer testers overall to appear slightly younger. An objective measurement of skin changes over time by application of the Visia^®^ imaging technology was found to be possible.

## Zusammenfassung

**Einleitung:**

Objektive Analyse der Effekte einer Serie von Hautpflegeprodukten durch Anwendung moderner Bildtechnologie.

**Methode:**

Die Studie wurde mittels 25 Freiwilliger durchgeführt, die eine plastisch-chirurgische Klinik besuchten. Die Kosmetika, die für die Untersuchung ausgewählt wurden, kamen von der Linie IMAGE MD^®^, die durch die Firma IMAGE Skincare zur Verfügung gestellt wurde. Bildaufnahmen der Gesichter wurden mit Hilfe der Visia^®^ Kamera der Firma Canfield Scientific aufgenommen. Die Freiwilligen stoppten ihr eigenes Hautpflegeregime nach den initialen Bildaufnahmen der Gesichter und wandten die Produkte über drei Monate an. 19 Freiwillige kehrten zurück und unterzogen sich erneut den Bildaufnahmen des Gesichtes. Acht verschiedene Hautaspekte – Flecken, Falten, Hauttextur, Poren, UV-Flecken, braune Flecken, rote Areale und Porphyrine – sowie das Hautalter wurden bestimmt und analysiert.

**Ergebnisse:**

Insgesamt erschienen die Freiwilligen um 1,4 Jahre jünger, allerdings nicht auf signifikantem Niveau. Es gab eine Streuung der Ergebnisse mit einer stärkeren Reduzierung des gemessenen Hautalters bei Frauen unter 55 Jahren um 3,2 Jahren. Die besten Effekte der untersuchten Produktlinie wurden für die Hautaspekte rote Areale und braune Flecken erreicht. Für die Kriterien Flecken, Hauttextur, Poren und UV-Flecken waren wenigstens zwei Drittel der Vergleiche zwischen den zwei Zeitpunkten, vor und nach der Behandlung, positiv. Für Falten und Porphyrine verbesserten sich die Ergebnisse nicht. Insgesamt verbesserte sich die Mehrheit der untersuchten Hautkriterien.

**Schlussfolgerung:**

Die Produktlinie IMAGE MD^®^ ließ die Freiwilligen insgesamt etwas jünger wirken. Eine objektive Messung von Hautveränderungen über die Zeit durch die Anwendung der Visia^®^-Bildaufnahmetechnologie wurde als durchführbar ermittelt.

## Introduction

The cosmetic industry has long been used to advertise their products based on various sales tools [1]. Frequently, subjective reports – so called client testimonials – of the quoted positive effects were cited, and high-shining images of the products were used. The American company Procter and Gamble became well known as a pioneer in brand management (https://us.pg.com/pg -history). It was one of the first companies that started to investigate the effects of their products in a more systematic way. Nevertheless, there was an overall historical paucity of scientific publications by the cosmetic industry into the true effects that were proposed. Up until the modern day there is still a lack of controlled studies regarding the efficacy of dermocosmetic products and the concept of evidence-based cosmetics was seen to have been only partly developed [2]. There remains a lack of evidence to support the claimed effectiveness [3] even though today there is an increasingly urgent need for objective investigations into these effects. 

**Figure 1:**
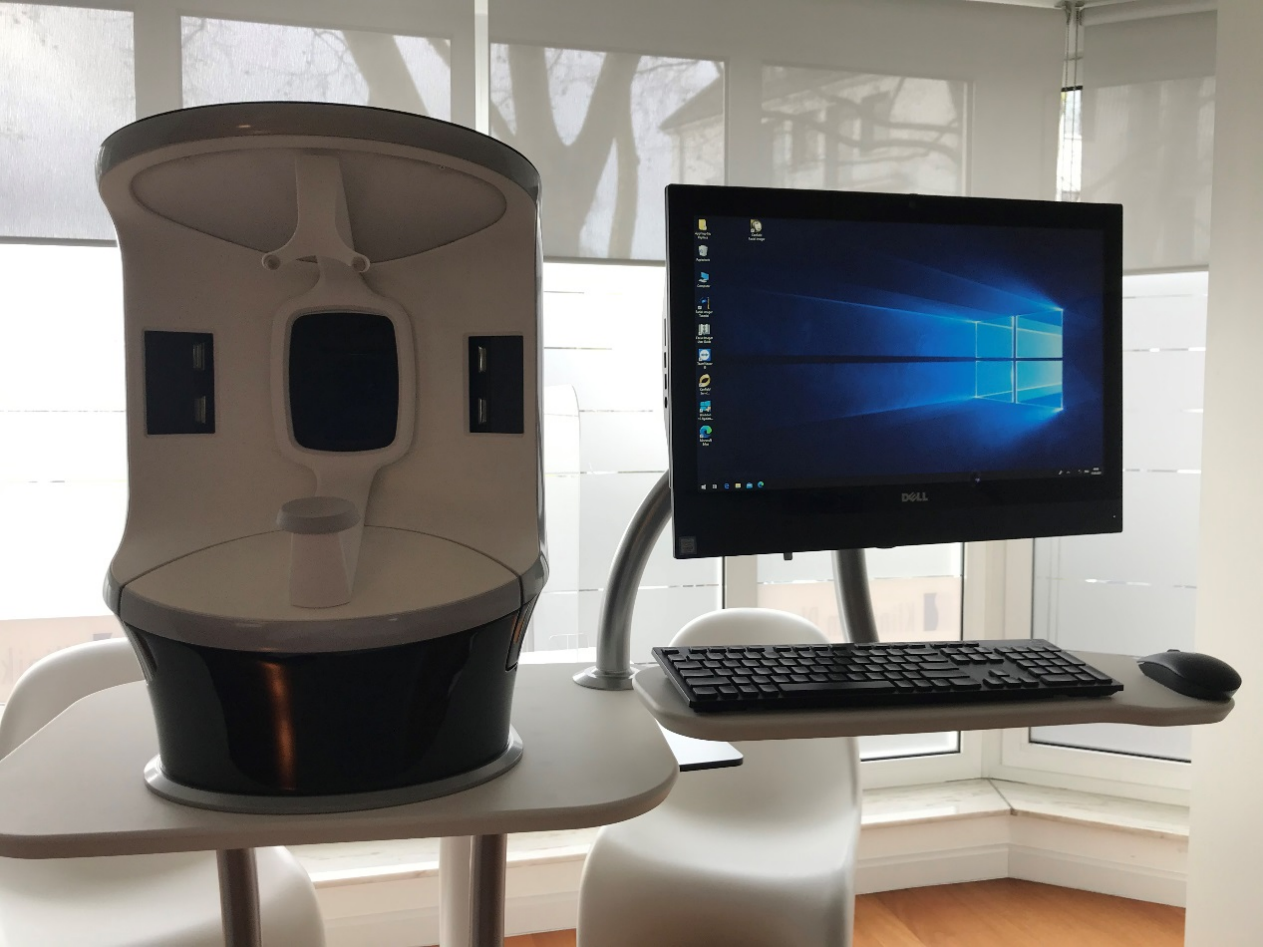
Visia^®^ complexion analysis camera system

One method to objectively investigate the condition of the skin and the possible effects that cosmetical products might have was made possible by modern digital imaging technology. The development of computer sciences created new options to visualise and analyse various skin criteria. In this context, the Visia^®^ camera system was developed by the American company Canfield Scientific Inc., New Jersey, USA (https://www.canfieldsci.com). The Visia^®^ is a commercialised clinical imaging device for facial capture and analysis (Figure 1 (Fig. 1)). As is the case with other technical systems, continued improvements were implemented which led to repeated updates of the previous camera systems. Accordingly, while the Visia^®^ was initially developed predominantly as a sales tool, today more options are on offer. The Visia^®^ camera system is a two- and three-dimensional high-resolution digital imaging tool for the visualisation and analysis of skin surface aspects. The first clinical applications in the circumscribed field of facial pore analysis and acne scaring have been reported [4], [5], [6].

Among the possible applications is the use of the Visia^®^ camera system as an objective analysis tool of various follow-up effects of treatments of cosmeceuticals. Despite this, to date there was a scarcity of publications of the Visia^®^ being used for this purpose. 

The American company IMAGE Skincare (https:// imageskincare.com) realised the potential offered by an objective outcome study of their own cosmeceutical products and had enough faith in their own products to undergo an independent investigation of it’s IMAGE MD^®^ line.

## Methods

An objective investigation into the effects of a cosmetic product line was conducted. The null hypothesis was that there was no provable statistical difference of the sample group at two time points, such as before and after having received a standardised cosmetic treatment – or that a connection was not provable from statistical point of view. If the probability of the error of the corresponding test was smaller than 5% (<0.05), then statistical significance was taken to exist and the null hypothesis was rejected. In this case, a difference that was found could be assumed to exist not only for the sample, but for the whole population. 

The volunteers to test the cosmeceutical products were recruited by notifying them of the planned study on the website of the local plastic surgical clinic, as well as by e-mail information sent out to the previous patients of the clinic. Participation was purely voluntarily and information regarding the participation was provided prior to the inclusion into the study. 

Patients who expressed their interest were invited to a personal consultation with a plastic surgeon and informed about all aspects of the study in verbal and written form. A written consent was obtained. Volunteers were provided with a series of cosmeceutical products by the company IMAGE Skincare and offered the latest development of the available products, the IMAGE MD^®^ line. Cosmeceutical products of this company used in this study were already used to be sold in the United States. There were four test products by the IMAGE MD^®^ line such as the IMAGE MD^®^ restoring facial cleanser, the youth serum, the daily defense moisturizer SPF 50 and the youth repair crème. The method of application following a standardised protocol was explained to the volunteers, with a scheme of application in the morning and evening. In case of problems, patients were able to contact the plastic surgical clinic and the principal investigator throughout the study. 

Inclusion criteria were women who had to agree to stop the own previous skin routine and exclusively use the MD^®^ products for three months. Facial capture with clean skin was planned before and after usage. 

Exclusion criteria were women who did not wish to stop their own cosmetics or did not wish to return for the follow-up captures, or who presented with acute health problems, acute skin problems, chronic skin diseases or known allergies. 

Facial images were taken with the Visia^®^ camera system by the company Canfield Scientific from the left, front and right aspect using different flashes to display the various superficial skin aspects. Objective data by the Visia^®^ camera to eight different skin aspects such as spots, wrinkles, skin texture, pores, UV spots, brown spots, red marks and porphyrins as well as to the skin age were obtained and analysed. 

Right after the initial facial capture, the IMAGE MD^®^ skincare treatment was started per the standardized protocol. Participants discontinued their previous skincare products and only applied the products from the IMAGE MD^®^ series until the end of the study, right up to the second capture with the Visia^®^ camera. Participants provided their demographic information, and the grading of their skin types according to the Fitzpatrick scale was done by automated software calculations by the Visia^®^ camera and subjective assessments.

Data were recorded as „percentiles“, „feature counts“ and as „absolute scores“ by the Visia^®^ camera. For the data of the percentiles a rise in the values was an improvement, whereas for the data of the feature counts and the absolute scores a fall in the values was an improvement.

All statistical evaluations were taken with the program R (R core Team 2016). R is a language and environment for statistical computing; R Foundation for Statistical Computing (https://www.R-project.org). All images were created with the package „ggplot2“ of the program R. All variables were considered to be interval-scaled and continuous and showed distances between them that were constant.

## Results

Out of the 25 women included in the study, who underwent the initial capture and started with the products, 19 completed the study and returned for follow-up capture. There were two drop-outs who preferred to continue daily skin care routine with their own previous skin care products instead of the MD^®^ series. Four women did not return for capture for undisclosed reasons. The youngest participant was 39 years of age and the oldest 68 years of age with a mean of 56 years among those who returned for the capture. 

**Figure 2:**
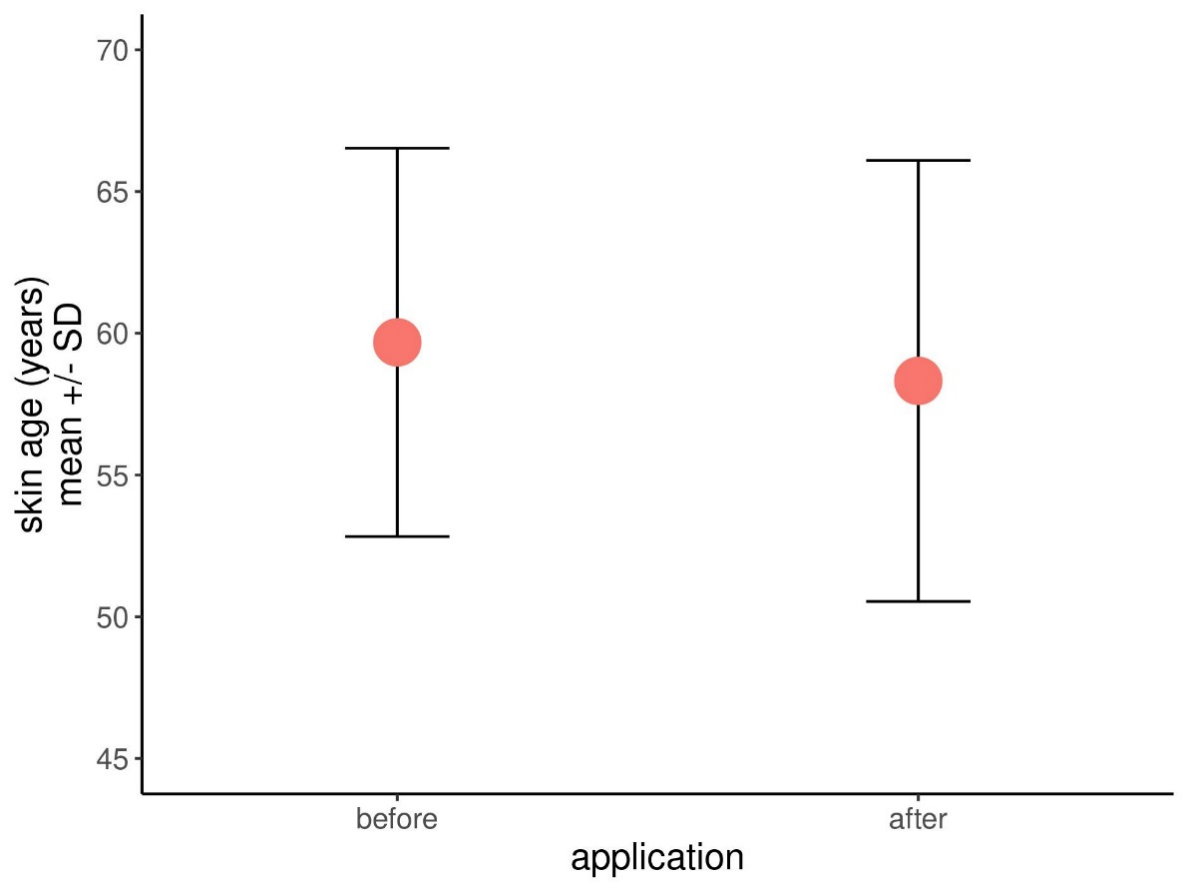
Results of the true skin age before & after application of the cosmetics

The measured true skin age fell after three months of using the product line from a mean of 59.7 to 58.3 years, with a standard deviation of 6.85 and 7.78 accordingly. Therefore, overall the volunteers turned out to appear younger by 1.4 years (Figure 2 (Fig. 2)). The difference was however not statistically significant at a p=0.165. 

**Figure 3:**
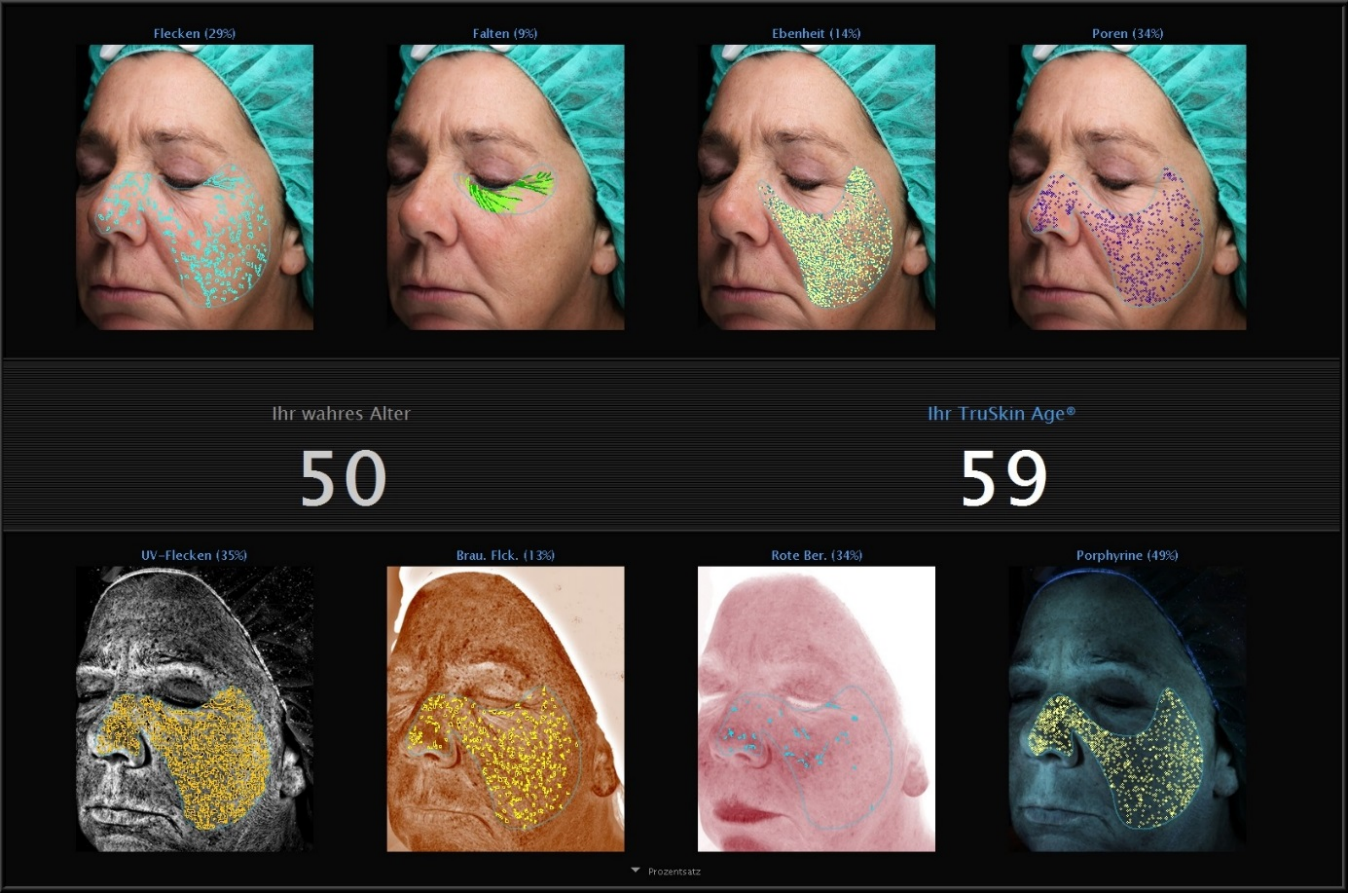
Example of one volunteer before application of the products

**Figure 4:**
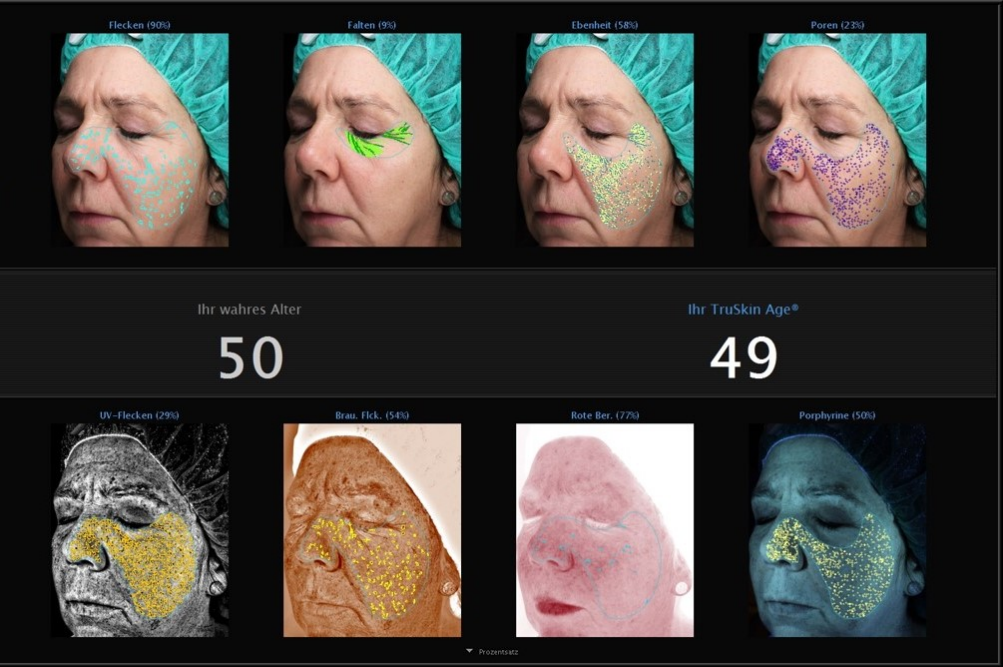
Example of one volunteer after application of the products with very good results

**Figure 5:**
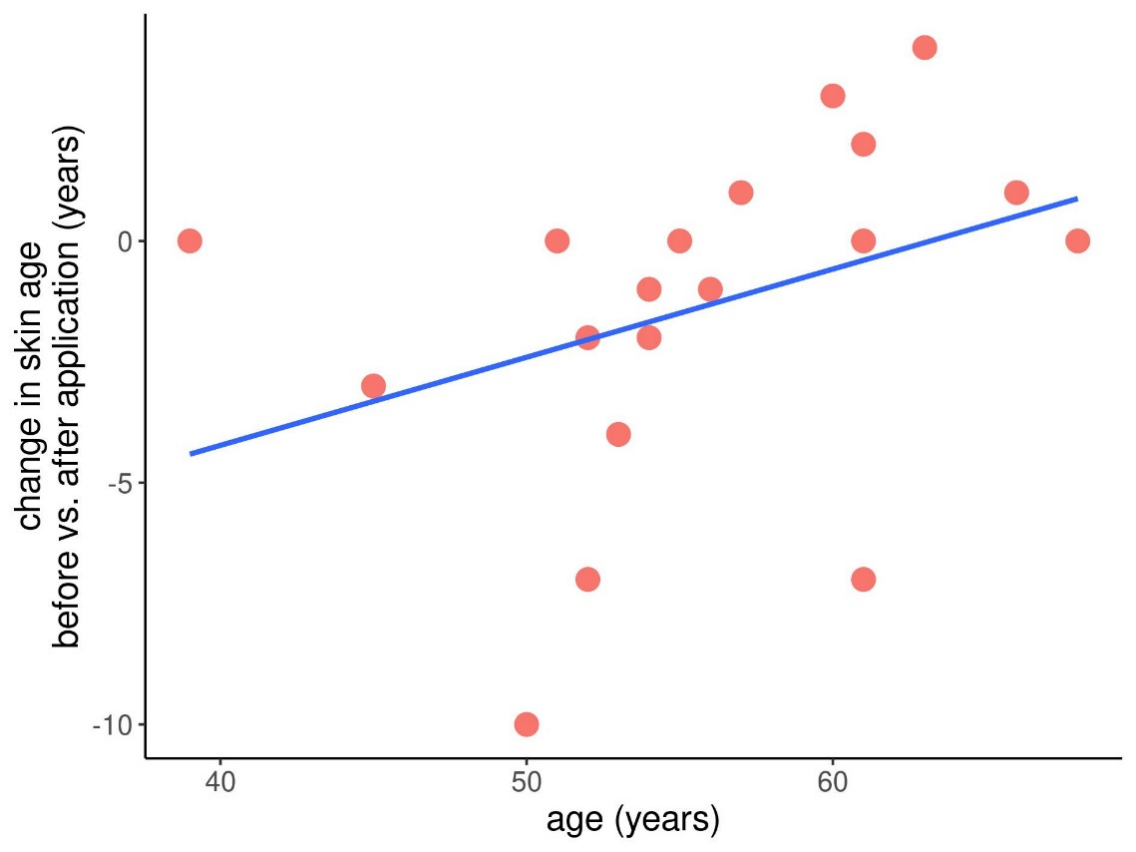
Spread of results in view to the change in skin age before and after application

There was a spread in the results with some volunteers to become younger (Figure 3 (Fig. 3), Figure 4 (Fig. 4)), some staying the same and some appearing older regarding the calculated true skin age (Figure 5 (Fig. 5)). 

When looking just at the women below the age of 55, none saw an increase in age, but there was a tendency of a stronger reduction in the measured skin age. These women turned out to appear younger by 3.2 years (spread: –10 to 0 years). These results, however, were again not statistically significant at a p=0.127 with a correlation coefficient by Pearson of R=0.36. 

When looking at the data of the true skin age as well as the eight single skin criteria, then in 71.2% of the comparisons between timepoint 1 and 2 there was a better result at timepoint 2, whereas the opposite was true in 28.8% of the cases. This result was obtained by calculation of 73 comparisons from the true skin age and the eight criteria from all three angles of capture for the 19 volunteers. 25 out of the 73 comparisons were calculated as statistically significant (p<0.05). These were all positive with the conditions at timepoint 2 being better than at timepoint 1. For the left perspective, 17 out of 24 comparisons were positive (70.8%) and 6 out of 24 were statistically significant, for the front perspective 18 out of 24 were positive and 9 significant, and for the right perspective 16 out of 24 were positive and 9 significant (Table 1 (Tab. 1)). 

Best effects of the studied product line were obtained for red marks and brown spots (Table 2 (Tab. 2)). In red marks, all nine comparisons were considered as statistically significant positive. These nine comparisons were obtained for the three specified parameters by the Visia^®^ camera, such as percentiles, feature count and absolute score recorded from three perspectives – left, front and centre – before and after application of the cosmeceuticals. 

Additionally, in brown spots 9 out of 9 comparisons were positive and 8 of these 9 comparisons were statistically significant. 

For the criteria spots, texture, pores, and UV spots, at least two thirds of the comparisons between the two time points were positive, however at a slightly lower significance level than in red areas and brown spots. 

In wrinkles and porphyrins, the therapy was in contrast less successful, with 2 out of 9 comparisons positive in wrinkles, and 0 out of 9 comparisons in porphyrins. Demographic data of the study group are provided (Table 3 (Tab. 3)).

**Figure 6:**
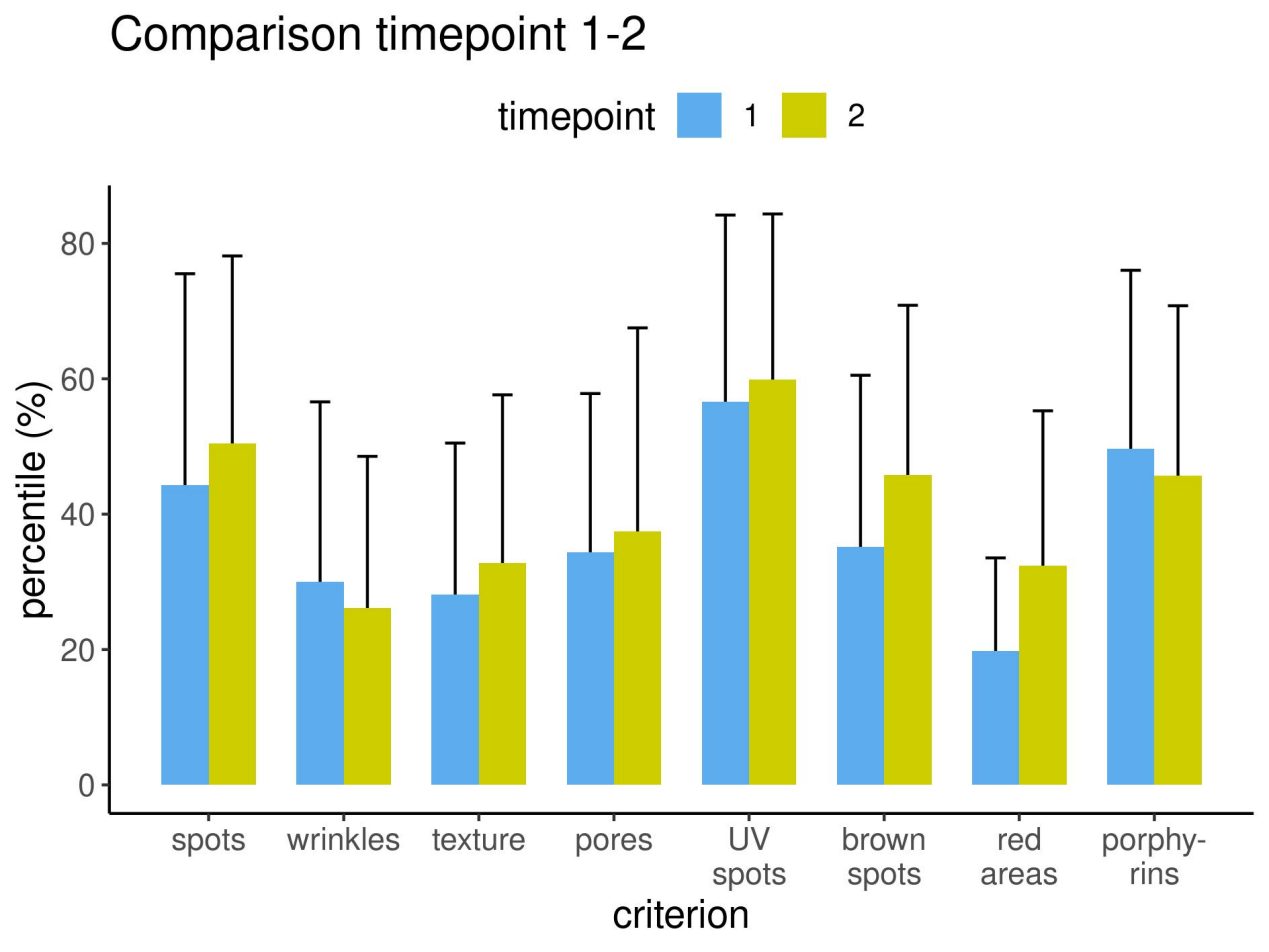
Results measured as percentile for each criterion before and after treatment

**Figure 7:**
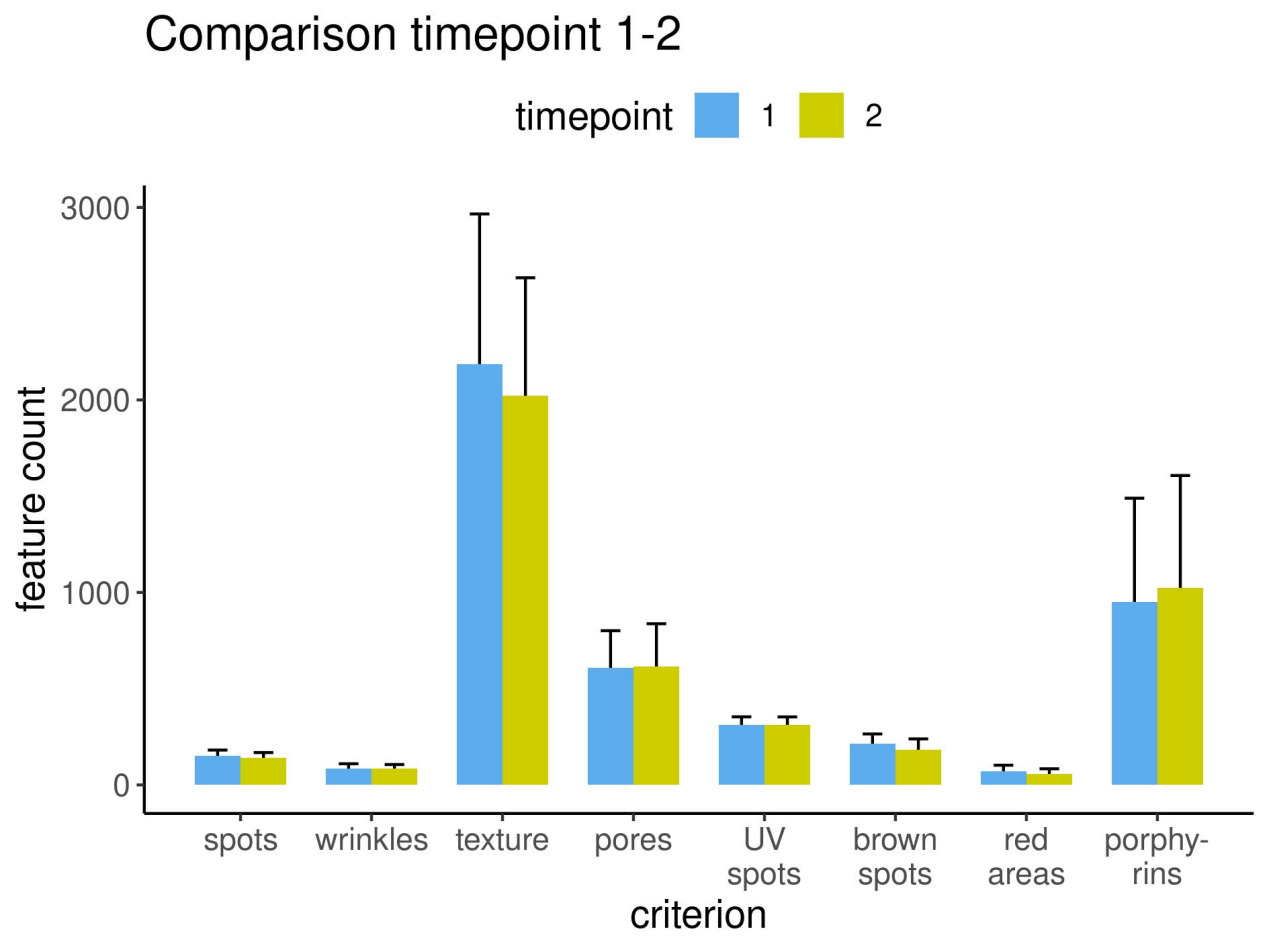
Results measured as feature count for each criterion before and after treatment

**Figure 8:**
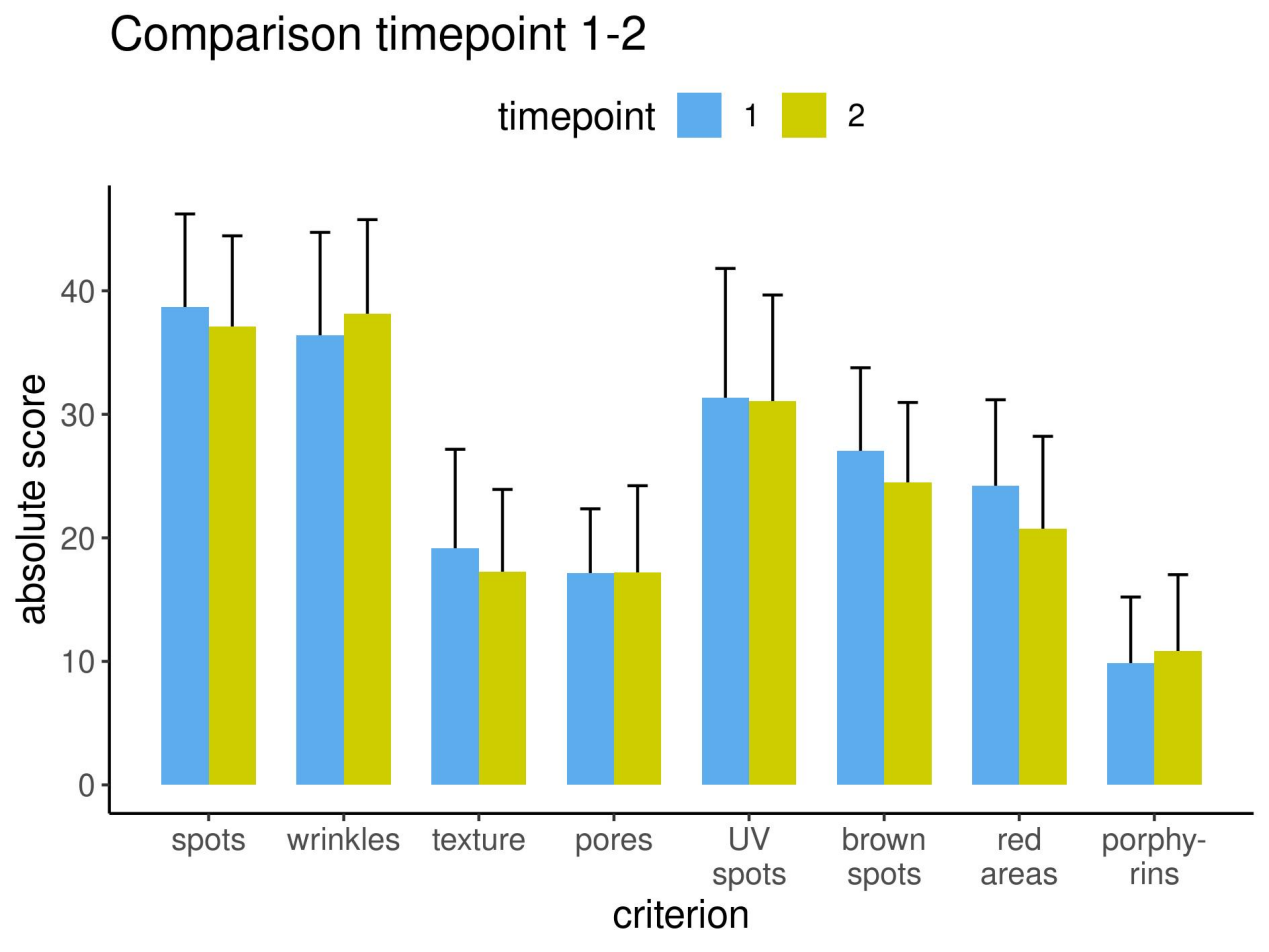
Results measured as absolute score for each criterion before and after treatment

The results from the left capture perspective are displayed for all eight criteria before and after treatment for the three measurement methods: percentile (Figure 6 (Fig. 6)), feature count (Figure 7 (Fig. 7)) and absolute score (Figure 8 (Fig. 8)). 

## Discussion

The results of this study revealed that it is possible to determine which aspects of the skin surface might benefit from a certain cosmetic product or not. In our case, the MD^®^ line gave excellent results in red areas and very good results in brown spots, yet presented hardly any results in wrinkles and no results in porphyrins. Furthermore, rejuvenation effects showed a better tendency to be obtained in the younger age group under 55 years than in the older women. This accrued knowledge gives rise to the possibility of advertising cosmetic products to reflect true measurable benefits. The study paves the way for a method that moves beyond mere promises and belief, which until now has seemed to dominate the advertisement of cosmetic products.

The study presented a longitudinal comparison between two timepoints, such as before and after the specified skincare treatment, within the same group of study participants. The question might arise if a comparison with a second group that did not use the same treatment might have been beneficial. However, a second group would present different demographic factors, different ages, and different history of smoking, diseases, or medication than the group under investigation so that errors that might distort the results could be induced. One way to reduce those errors would be to raise the number of participants for comparison, but even in this case, the demographic factors still must be assumed to be quite different. Another study design would focus on a separate research question and lead to different results accordingly. However, from a statistical point of view, it is preferable and more reliable to draw a comparison within the same group when a treatment effect is under investigation. This increase in reliability is because all other possible factors that might influence the measurements are kept the same; only the aspect of the unique and standardized treatment under investigation is changed. The skin analysis in the initial capture is the starting point of a “no treatment” situation, as no treatment was provided beforehand, compared to the image captures in due course and the objective skincare analyses after treatment. Therefore, data is compared between timepoints 1 and 2.

While traditional cosmetical products remain without sound objective validation studies, the cosmetic industry continues to be among the fastest growing industries in the last decade. Moreover, the new terms of ‚cosmeceuticals‘ and ‚nutricosmetics‘ have been created to imply possible health benefits [7]. It was found however that in nutraceuticals and cosmeceuticals, which are considered as cosmetics that promise benefits without the incorporation of prescription drugs [8], there seems to be only minimal regulation [9], [10].

The study here provides a good example to illustrate some discrepancy between desired and measurable effects of a cosmetical skin care regime. Measurements were conducted with what is deemed to be the best and most detailed skin imaging tool on the market, according to the manufacturers of the Visia^®^ camera. Nevertheless, while this study was conducted in a prospective manner and followed a standardised protocol, the measured changes in fact showed mixed results; some aspects improved, other however did not. Reasons for this can be varied and remain a subject for discussion. The providers of the MD^®^ skin care product series that was investigated in this study voiced great belief not only in the quality of their own products but also in their capability to significantly improve skin conditions. However, the truly measurable changes were less pronounced than desired, despite an overall improvement in the majority of the examined skin aspects. 

Interestingly, the study showed that it was the red areas and brown spots that improved the most after application of the MD^®^ skin cosmetic series over time. A new technology developed by the company Canfield for the Visia^®^ camera, RBX, served especially in the red and brown spectrum, to such an extent as to visualise skin conditions related to vascular disorders as well as hyperpigmentation [11]. Hence the strength of the Visia^®^ camera might have contributed to the detection of the changes in the red and brown aspects of the skin surface. Consequently, an area for further discussion is whether the detection of mixed improvements in the other skin aspects might instead have been due to the measurement method rather than any truly limited effects of the IMAGE MD^®^ skin care products. 

Further possible reasons for the results remain to be discussed. One such reason might be that it is simply attributable to skin anatomy. When investigating the principles of skin anatomy and physiology, it is clear to see that the top layer of the skin – the epidermis – consists of unperfused, non-living skin cells. It is therefore understandable that, when applying cosmetic products on top of this epidermal layer, the exact result of skin improvements cannot necessarily be anticipated. While cosmetic companies have worked for many years on the back of extensive promises regarding the possible effects of their products, in actuality the true measurable effects seem in fact to remain less pronounced. This might be one of the reasons why to the present day there are only limited studies that try to measure these effects in an independent and objective way. The industry still relies on reports of individual experiences of some happy customers or celebrities to increase their sales, instead of conducting costly, time-consuming, prospective and independent studies. All the while, aging has been shown to be a complex process, and skin appearance is just one aspect of it.

A company that tries to meaningfully establish the effects of the own product range should therefore be recognised for this effort. Interestingly, in this regard there seem to be some cultural differences between the countries; while some volunteers in our studies mentioned some dissatisfaction with the skin product series in the beginning of their usage, similar objections were not reported by the company in the United States, where the products are sold with great success. Reasons for this might be that expectations were not met and that the composition of European skin care regimes might be softer and less exfoliating in comparison to the products of the American company investigated here for skin rejuvenation. 

From a statistical point of view, the type of analysis presented here was explorative. A whole range of parameters were collected and examined in several directions. From the data, an impression is gained in which direction the IMAGE MD^®^ treatment of the skin can show effects. Based on this data, it becomes possible to pick one aspect of interest in a future study and formulate a hypothesis for further investigation, given the treatment effect that could be confirmed or rejected. 

Overall, the options the Visia^®^ camera offered in a view to presenting an objective skin analysis were exciting and open up a number of applications in the future. As a limitation for the application emerged that the researchers felt that the Visia^®^ camera system relied on a somewhat steep learning curve regarding the details of the usage. 

There is a general need to investigate cosmetic outcomes objectively and quantitatively [12]. The Visia^®^ camera system presents one such option to achieve this.

## Conclusion

The IMAGE MD^®^ product series caused the volunteer testers overall to appear slightly younger when analysed by the Visia^®^ camera system, with a tendency of women under 55 years of age to benefit the most.

Best improvements of skin appearance were seen in red areas and brown spots, with excellent results for these aspects. In contrast, concerning wrinkles and porphyrins hardly any effect was found. Positive results in two thirds of the cases were found in spots, texture, pores and UV spots. 

An objective measurement of skin changes over time by application of the Visia^®^ imaging technology was found to be possible.

## Notes

### Acknowledgement

I thank Dr. H. Hofheinz for his support for the study and in searching the volunteers.

I thank the staff of our clinic to help organise the visits of the volunteers to the clinic. I thank Dr. M. Ronert for the provision of the „IMAGE MD^®^ clinical skincare“ products to conduct the study. 

I thank Dr. Wolfgang Reimers for his statistical advice in the planning of the study and his support in the analysis of the data. 

### Ethical statement

All procedures performed in the study were in accordance with the ethical standards of the institutional and national research committee and with the 1964 Helsinki declaration and its later amendments or comparable ethical standards.

### Competing interests 

The author declares that she has no competing interests. There was no influence of the companies involved into the conduct of the study, nor in the analysis or the publication of the results. 

## Figures and Tables

**Table 1:**
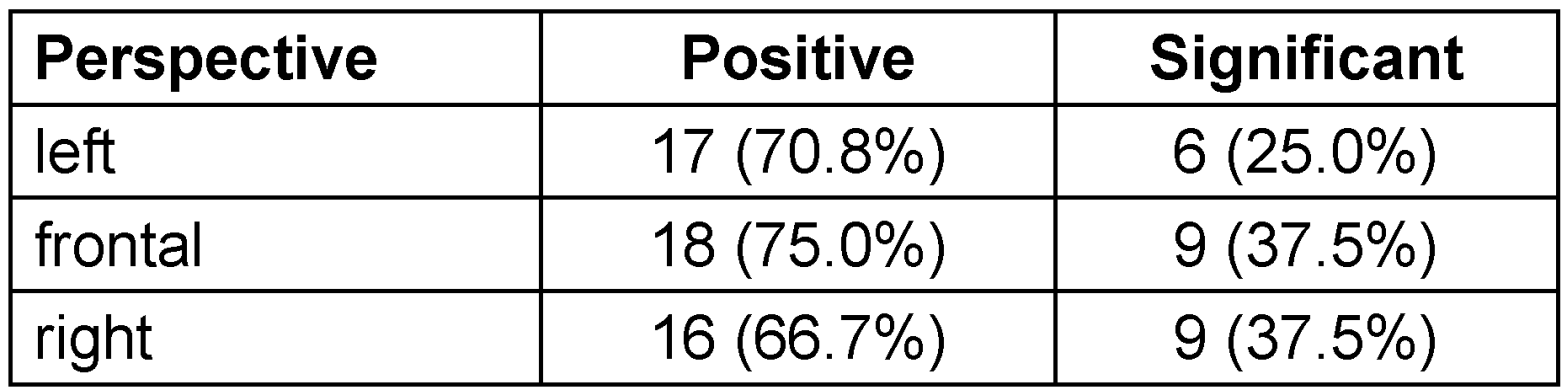
Positive and significant changes for all criteria for the three capture perspectives

**Table 2:**
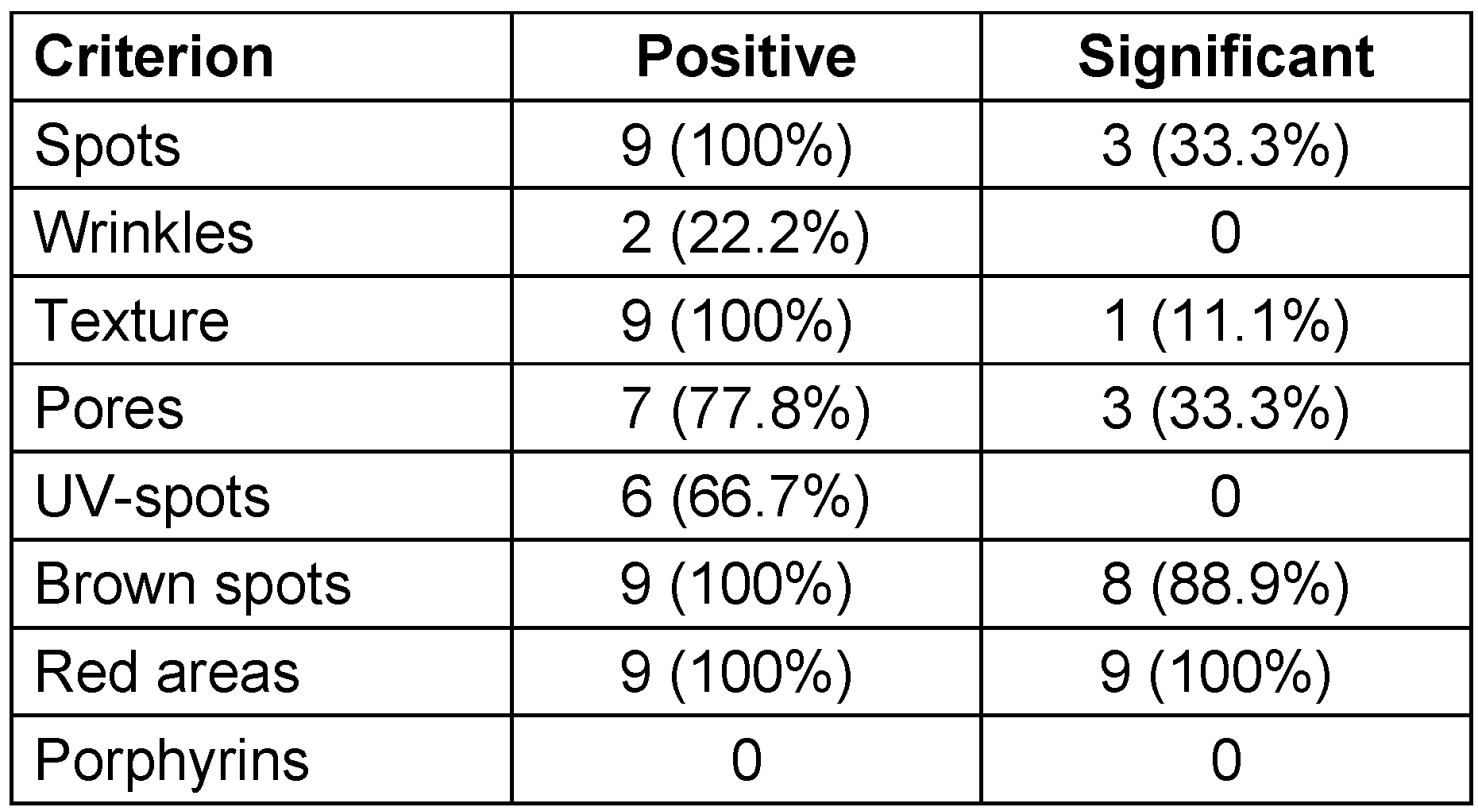
Positive and significant changes for each of the eight criteria

**Table 3:**
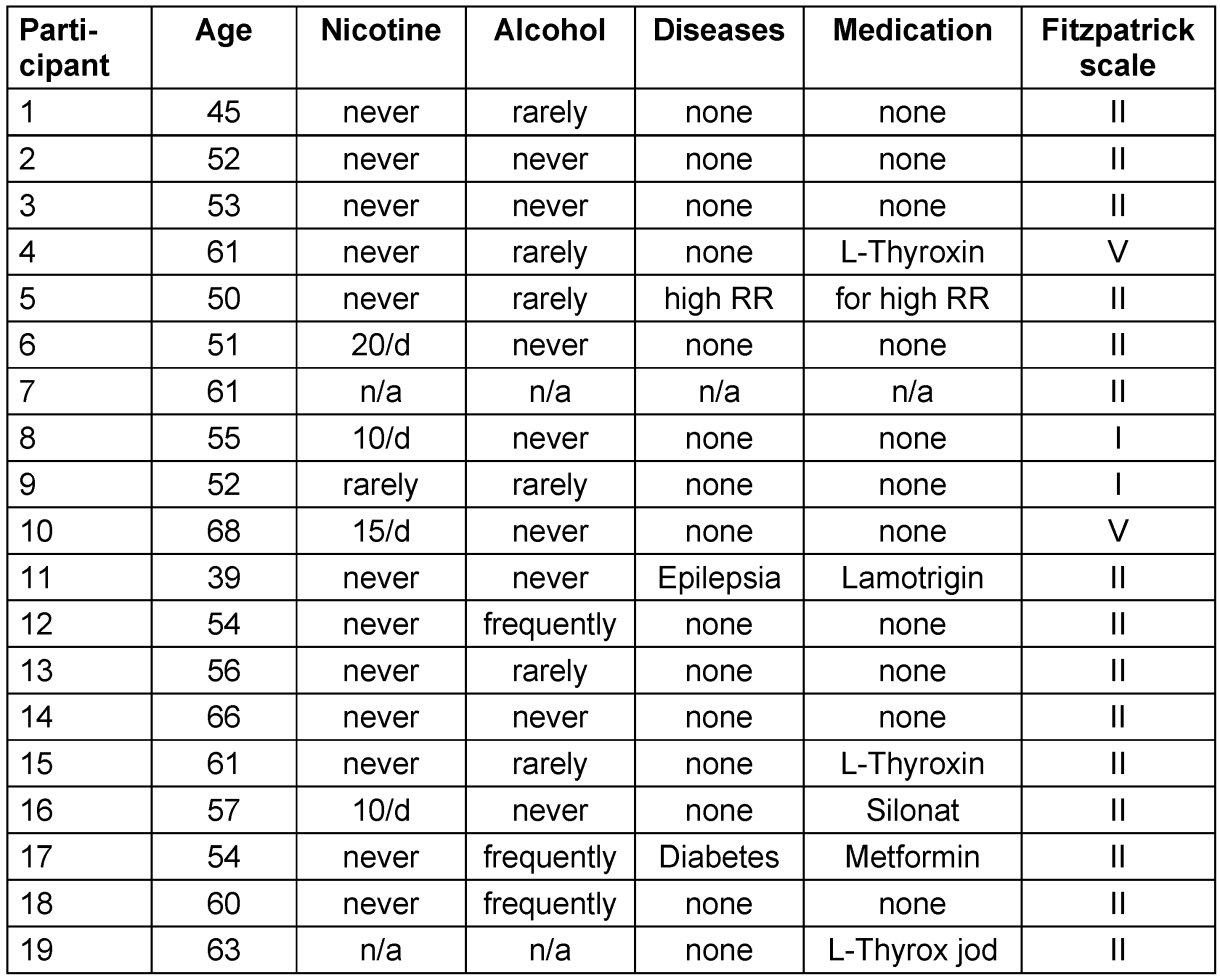
Demographic data of study group
